# Strategies to improve enrollment in The Special Supplemental Nutrition Program for Women, Infants, and Children (WIC): examining high coverage states and leveraging successful COVID-19 pandemic adjustments

**DOI:** 10.1017/S1368980022001471

**Published:** 2022-07-22

**Authors:** Amanda G Kuhn, Karen G Ganacias, Janine A Rethy

**Affiliations:** 1 Georgetown University, School of Medicine, 3900 Reservoir Rd NW, Washington, DC 20007, USA; 2 MedStar Georgetown University Hospital/Georgetown University School of Medicine, Department of Pediatrics, 3800 Reservoir Rd NW, Washington, DC 20007, USA

**Keywords:** Food insecurity, COVID-19 adjustments, Low-income population, WIC enrollment, Access strategies

## Abstract

The Special Supplemental Nutrition Program for Women, Infants, and Children (WIC) is an essential program in the USA providing food benefits and nutritional and breast-feeding support to low-income pregnant or postpartum women, infants and children at nutritional risk. Despite similarities amongst federal regulations shared across WIC programs at the state level, important differences in the operations, policies and technologies between states exist. Nationally, nearly half of women, infants and children who were eligible to receive WIC benefits in 2018 were not participating in the program. In this paper, we evaluate common practices exhibited by states with the highest and lowest WIC coverage rates to identify strategies that may improve enrollment and retention rates in regions with low WIC coverage rates. We use WIC as a case study for identifying strategies that can be broadly applied to improve utilisation of similar food assistance programs globally, particularly those benefiting low-income women and children. The four strategies discussed here include utilising data to check adjunctive eligibility and reach eligible non-participants, increasing public awareness of WIC through outreach and referral efforts, implementing a centralised smartphone app and linking personal electronic benefits and streamlining the use of technologies for online applications, participant portals and remote communication. In most states, the COVID-19 pandemic and the federal waivers issued in response have offered the opportunity to promptly implement some of these strategies, particularly with regard to remote communication capabilities. With proper resources and implementation, these strategies can improve utilisation of WIC and similar programs globally.

Established in 1972 through the US Department of Agriculture’s (USDA) Food and Nutrition Service, Special Supplemental Nutrition Program for Women, Infants, and Children **(**WIC) is a food and nutrition assistance program benefiting low-income women, infants and children up to 5 years of age who are at nutritional risk, such as anaemia or under-weight^(1)^. In the 2020 fiscal year, WIC served over six million women, infants and children per month, including nearly half of US-born infants^(2)^. Benefits of the program include food, nutrition and breast-feeding support, immunisation screening and health and community referrals. Federal grants are provided to states, and state agencies manage the program according to specific federal regulations, as well as direct the operations of local agencies, where WIC services are provided^(3)^.

Many who are eligible to enroll in WIC do not participate in the program. According to national data from the US Department of Agriculture’s Food and Nutrition Service in 2018, the average coverage rate in 2018 for all eligible participants was 56·9 %, meaning nearly half of those who were eligible were not participating in WIC^(4)^.

In this commentary, we examined states and provinces with the highest (Vermont, California, Minnesota, Maryland and Massachusetts) and lowest (Montana, Utah, District of Columbia, New Hampshire and New Mexico) WIC coverage rates according to these data to identify patterns of strategies and operational practices that may be contributing to higher enrollment and retention^(4)^. While there is no single aspect that explains the disparities in coverage rates at the state level, and not all of these strategies are completely feasible in all regions, specific combinations of these practices could likely improve WIC coverage in regions with low coverage rates. Of note, some of these strategies build on those previously proposed by The Center on Budget and Policy Priorities for improving aspects of WIC eligibility and enrollment^(5)^. This paper provides additional strategies that our literature review reveals are common in high-coverage WIC programs, including lessons learned from the COVID-19 pandemic, that are applicable for similar existing or piloted food assistance programs in other countries. For one such program in the U.K., Healthy Start, nearly 30 % of eligible families were not enrolled in the program as of March 2022^(6)^, highlighting the need to improve the utilisation of programs globally.

## Strategies

### Streamlined process for confirming adjunctive eligibility between assistance programs and proactively identifying eligible participants

States that have high-level capacity for an adjunctive eligibility check between assistance programs decrease barriers to enrollment in more than one program. According to federal regulations, an individual who is enrolled in other assistance programs, such as the Supplemental Nutrition Assistance Program, Temporary Assistance for Needy Families or Medicaid are adjunctively income-eligible for WIC^(7,8)^. To prove income eligibility, most states accept documentation from one of these programs and also have at least one alternative method of checking adjunctive eligibility, such as through online program portal data or an automated phone call^(9)^. All five states with the highest coverage rates in 2018 have long utilised one or more of these alternative methods, while some states with the lowest coverage rates have implemented these capabilities within more recent years (N.M. and N.H.)^(5,9,10)^. More uniquely, two of the five highest coverage states can assess for adjunctive eligibility through an interface that is part of their WIC eligibility system (C.A. and M.D.)^(9)^.

These methods can also be used to identify adjunctively eligible nonparticipants, or those who are enrolled in a qualifying benefit program but are not enrolled in WIC. For example, Vermont has a monthly report that lists people who are currently enrolled in Medicaid but not in WIC, and WIC staff contacts those people to set up an appointment, if desired^(11)^. Data were collected on these efforts, and 63 % of those who could be reached by phone scheduled an enrollment appointment^(11)^. A similar approach with texting was piloted in four states, including Virginia, where it was found that those enrolling all adjunctively eligible Virginia families in WIC would decrease the current coverage gap from 58 % of WIC-eligible individuals not currently enrolled to 18 %^(12)^. While agencies could face challenges with incompatibility between information systems or obtaining data sharing agreements, this is a powerful strategy that can decrease barriers to enrollment and proactively reach people who would likely benefit from WIC.

### Increasing awareness of program availability through outreach, healthcare provider referrals and other community resources

While not necessarily a novel approach, there is an area of under-tapped potential in leveraging healthcare providers in improving WIC coverage. According to interviews of a representative sample of WIC participants in the Second National Survey of WIC Participants, 61 % of first-time WIC participants with other children reported not previously participating in WIC due to not being aware of the program^(13)^. Increasing paediatrician involvement in referring eligible nonparticipants can help improve coverage rates for children, especially as coverage rates are lowest for children and drop off with increasing age^(4)^. Though there are many items to address during a single well-child visit, community paediatricians can play an integral role in this referral process, especially by referring families participating in Medicaid. A WIC provider referral form is available in many states and provinces, though a recently conducted Washington, D.C., provider survey suggests that only a small percentage of providers participate in the WIC referral process, and most did not feel adequately prepared to educate effectively (Ganacias 2022, unpublished results). All five states with the top WIC coverage rates have sophisticated websites containing in-depth resources and referral forms for medical professionals^(14–18)^. In lower coverage states, the websites are less complete and user-friendly. Provider knowledge of or utilisation of these available online resources is also an important factor (Ganacias 2022, unpublished results).

Community outreach, through partnerships or social media, is also an area for growth. Approximately half of local agencies surveyed in the Third National Survey of WIC Participants offered certifications at alternative sites, such as offsite clinics or schools, as of 2019^(19)^. Many agencies have focused on enhancing collaboration with early childhood intervention programs such as Head Start through coordination and co-location of services, data sharing and other strategies^(20)^.

### Implementing a centralised smartphone app and linking personal electronic benefits

Historically, WIC participants have been issued paper WIC checks that they need to pick up each month from the WIC office and take with them to WIC stores. An Electronic Benefits Transfer (EBT) card serves as a debit card at WIC-participating grocery stores for purchasing WIC-eligible food items. Currently, forty-eight states have implemented statewide EBT benefits for WIC in response to a federal mandate, and the remaining are in the process of implementing or piloting EBT cards^(21)^. According to the 2014 Arizona WIC Program Child Retention Project, completed prior to EBT implementation in Arizona, 70 % of women who were previous participants of WIC reported they would return to WIC if there were improvements to the shopping experience, specifically if an EBT card was used, shopping for WIC foods was easier, or the grocery store experience was less embarrassing^(22)^. Further, a recent study found that offline EBT reissuance practices, in which one must mail in or physically present an EBT card for reloading of benefits, was associated with a significant relative decrease in WIC participation during COVID-19 as compared with online, or remote, reissuance practices^(23)^.

A WIC smartphone app is the next generation of the digital EBT concept. With the ubiquity of smartphones, an app can improve retention and utilisation and may include functionality to easily check benefits status, access WIC information and identify WIC-eligible items in a store. Of the top five coverage states, California, Minnesota and Maryland have state-specific WIC smartphone apps, and Massachusetts and Vermont utilise either the partial or full capabilities of the WIC Shopper App, developed by JPMA, Inc.^(24)^. Approximately thirty-one states or provinces utilise this app, though some states or provinces do not utilise its full capabilities. In addition to providing the WIC food list, closest WIC office or store and recipes or other education links, other features that are available with this app include a barcode option to scan an item and see if it is WIC approved and the ability to link EBT card information to check current benefits balance.

### Optimising the use of technology for remote communication capabilities, participant portals and online applications

In light of the COVID-19 pandemic, many WIC operations transitioned to remote communication via phone or video appointments, remote certification and remote benefits issuances through waivers instituted by the U.S. Department of Agriculture^(25)^. In a recent report, nearly all state agencies reported that federal waivers, specifically those waiving the physical presence requirement and enabling remote issuance of benefits, have improved WIC safety, accessibility and convenience for participants, though many also reported the transition to remote communication to be challenging^(26)^. Only 12 % of local agencies offered remote certifications before the pandemic compared with nearly every local agency during the pandemic^(26)^. Extending a telehealth option beyond these temporary waivers would reduce scheduling, childcare and transportation barriers to accessing WIC benefits.

The National WIC Association and Nava Public Benefit Corporation assessed online features implemented by WIC state agencies, many of which were employed during COVID-19 pandemic and offered recommendations for implementing these features at the state and local level^(27)^. According to this report, as of October 2020, just five of the eighty-nine state agencies use an online portal as a centralised WIC experience for use both during and after certification, three use a document uploader for uploading certification or physician’s documents and some agencies reported using automated chatbots to answer common questions and reduce call volumes^(27)^. A centralised online portal, such as that used in Michigan, helps participants to check benefits, find WIC-participating stores and manage appointments^(5,28)^.

Additionally, WIC staff reported that texting participants telehealth appointment reminders has increased show rates during the COVID-19 pandemic^(27)^. While the ability for people to receive information regarding documentation, nutrition tips or upcoming appointment reminders is helpful, offering options for bidirectional communication could be beneficial. Currently, four of the five highest coverage states allow applicants to send required documentation via text message or a texted upload link, and all five allow doing so via email^(9)^. While some agencies have a call-back feature if texted, bidirectional texting would make communication faster and more convenient^(29)^. However, only one-fifth of respondents in a National WIC Association survey of state and local agencies nation-wide in 2017 reported having capabilities for two-way texting^(30)^.

Finally, twenty-four of the eighty-nine state agencies have an online pre-application form that can be used to collect personal and contact information securely and possibly inquire about income eligibility or allow for uploading of documentation^(27)^. After information is submitted, WIC staff typically reach out to applicants to set up a WIC appointment. Four of the five of states with top coverage rates have some sort of online application tool (V.T., C.A., M.N., M.A.)^(31–35)^. Each of these methods can help increase WIC accessibility and streamline processes for WIC staff.

## Conclusion

The strategies discussed in this article, employed by many of the US states with the highest WIC coverage, can improve WIC’s accessibility, flexibility and ease of use to increase coverage and retention in regions with lower coverage. Furthermore, using WIC as a case study, these strategies can be applied globally to improve the utilisation of similar food and nutrition assistance programs. The discussed strategies are not an exhaustive list, and their utilisation by regions with higher coverage rates is not necessarily a measure of their contribution to higher coverage rates. Additionally, each strategy could take considerable time to implement and optimise for efficiency. The COVID-19 pandemic has exacerbated food insecurity globally, highlighted the need for highly functional food access programs and gave an unexpected opportunity for organisations to pivot towards more digitally enhanced solutions. For many organisations, it will be a strategic decision to permanently implement some of the policies and strategies employed during the pandemic to continue meeting the needs of women and children. Further research needs to be performed to evaluate the impact of individual strategies on closing the coverage gap, to expand the reach of WIC and similar nutrition programs worldwide and impact the health of low-income women and children.

## References

[1] U.S. Department of Agriculture Food and Nutrition Service (2021) Special Supplemental Nutrition Program for Women, Infants, and Children (WIC). https://www.fns.usda.gov/wic (accessed December 2021).

[2] U.S. Department of Agriculture Economic Research Service (2021) WIC Program. https://www.ers.usda.gov/topics/food-nutrition-assistance/wic-program/ (accessed December 2021).

[3] U.S. Department of Agriculture Food and Nutrition Service (2021) Special Supplemental Nutrition Program for Women, Infants, and Children (WIC) State Agency. https://www.fns.usda.gov/wic/state-agency (accessed December 2021).

[4] Gray KF , Mathieu KM , Johnson P et al. (2021) National- and State-Level Estimates of WIC Eligibility and WIC Program Reach in 2018 With Updated Estimates for 2016 and 2017. https://www.fns.usda.gov/wic/national-and-state-level-estimates-wic-eligibility-and-wic-program-reach-2018-updated (accessed December 2021).

[5] Neuberger Z (2017) Modernizing and Streamlining WIC Eligibility Determination and Enrollment Processes. https://www.cbpp.org/research/modernizing-and-streamlining-wic-eligibility-determination-and-enrollment-processes (accessed January 2022).

[6] Anonymous (2022) NHS Healthy Start Uptake Data. https://www.healthystart.nhs.uk/healthcare-professionals/ (accessed May 2022).

[7] U.S. Department of Agriculture Food and Nutrition Service (2020) WIC Eligibility Requirements. https://www.fns.usda.gov/wic/wic-eligibility-requirements (accessed December 2021).

[8] Anonymous 7 CFR Part 246 Special Supplemental Nutrition Program for Women, Infants and Children. February 13, 1985. Last updated March 11, 2022. https://www.ecfr.gov/current/title-7/subtitle-B/chapter-II/subchapter-A/part-246 (accessed April 2021).

[9] Neuberger Z (2022) State WIC Agencies Use Federal Flexibility to Streamline Enrollment. https://www.cbpp.org/sites/default/files/3-31-22fa.pdf (accessed May 2022).

[10] New Mexico Department of Health (2020) New Mexico WIC Policies and Procedures: Adjunctive Income Eligibility. https://www.nmwic.org/nm-wic-staff/policy-procedures/ (accessed January 2022).

[11] Vermont WIC Program (2019) Increasing Vermont WIC Program Enrollment: 2019 Report to the Legislature. https://humanservices.vermont.gov/our-work/reports (accessed January 2022).

[12] Maneely J & Neuberger Z (2021) Matching Data Across Benefit Programs Can Increase WIC Enrollment. https://www.cbpp.org/research/food-assistance/matching-data-across-benefit-programs-can-increase-wic-enrollment (accessed January 2022).

[13] Huang G , Geller DM & ICF Macro (2012) National Survey of WIC Participants II Volumes I–IV. https://www.fns.usda.gov/national-survey-wic-participants-ii (accessed January 2022).

[14] Anonymous Vermont WIC Resources for Health Professionals. https://www.healthvermont.gov/children-youth-families/wic/resources-health-professionals (accessed March 2022).

[15] Anonymous WIC Information for Health Care Providers. https://www.health.state.mn.us/people/wic/hcp/index.html (accessed March 2022).

[16] Anonymous WIC Information for Providers. https://www.mass.gov/service-details/wic-information-for-providers (accessed March 2022).

[17] Anonymous Women, Infants & Children (WIC): Health Care Providers. https://www.cdph.ca.gov/Programs/CFH/DWICSN/Pages/HealthCareProviders.aspx (accessed March 2022).

[18] Anonymous (2022) For Health Care Providers. https://health.maryland.gov/phpa/wic/Pages/wic-hcp.aspx (accessed March 2022).

[19] Magness A , Williams K , Garza A et al. (2021) Third National Survey of WIC Participants (NSWP-III): Brief Reports 1–10. https://www.fns.usda.gov/wic/third-national-survey-wic-participants (accessed January 2022).

[20] HHS, Office of Administration for Children and Families, USDA et al. (2019) Enhancing Participant-Centered Services Between the Special Supplemental Nutrition Program for Women, Infants, and Children (WIC) and Head Start Programs. https://wicworks.fns.usda.gov/resources/10-ways-wic-and-head-start-can-collaborate (accessed January 2022).

[21] U.S. Department of Agriculture Food and Nutrition Service (2021) WIC EBT Activities. https://www.fns.usda.gov/wic/wic-ebt-activities (accessed January 2022).

[22] U.S. Department of Agriculture WIC Works Resource System (2014) Summary of WIC State Agency Strategies for Increasing Child Retention. https://wicworks.fns.usda.gov/resources/summary-wic-state-agency-strategies-increasing-child-retention (accessed January 2022).

[23] Vasan A , Kenyon CC , Roberto CA et al. (2021) Association of remote *v*. in-person benefit delivery with WIC participation during the COVID-19 pandemic. JAMA 326, 1531–1533. 34415293 10.1001/jama.2021.14356 PMC8527357

[24] JPMA JPMA and WIC: WICShopper and WICSmart. https://jpma.com/ (accessed January 2022).

[25] Anonymous (2022) WIC COVID-19 Waivers. https://www.fns.usda.gov/programs/fns-disaster-assistance/fns-responds-covid-19/wic-covid-19-waivers (accessed March 2022).

[26] Lee H (2021) Changes in USDA Special Supplemental Nutrition Program for Women, Infants, and Children (WIC) Operations During the COVID19 Pandemic: A First Look at the Impact of Federal Waivers. https://www.fns.usda.gov/wic/operations-impact-federal-waivers-during-covid-19-pandemic (accessed January 2022).

[27] National WIC Association and Nava Public Benefit Corporation (2020) Supporting WIC Enrollment: Using Technology to Improve Certification Experience for WIC Participants and WIC Agencies. https://s3.amazonaws.com/aws.upl/nwica.org/wic-technology-landscape-_-final-report-design.pdf (accessed January 2022).

[28] Michigan Department of Health and Human Services (2021) Michigan WIC Client Connect. https://wiccp.state.mi.us/clientPortal/Default.aspx (accessed January 2022).

[29] South Los Angeles Health Projects WIC Program Text Messages for WIC Parents. https://wicforyou.org/wic-texts/ (accessed January 2022).

[30] National WIC Association (2018) WIC Outreach and Retention Survey Report. https://s3.amazonaws.com/aws.upl/nwica.org/outreach-and-retention-survey-report-2018.pdf (accessed January 2022).

[31] Vermont Department of Health Apply to WIC. https://www.healthvermont.gov/children-youth-families/wic/apply (accessed January 2022).

[32] PHFE WIC California Apply for WIC Online. https://www.phfewic.org/how-wic-works/apply-for-wic/ (accessed January 2022).

[33] San Francisco Department of Public Health How to Apply for WIC? https://www.sfdph.org/dph/comupg/oprograms/NutritionSvcs/WIC/howtoapply.asp (accessed January 2022).

[34] Minnesota Department of Health Apply for WIC. https://redcap.health.state.mn.us/redcap/surveys/?s%20=%20LNKN377EPE (accessed January 2022).

[35] Massachusetts Department of Public Health Apply for WIC online. https://www.mass.gov/forms/apply-for-wic-online (accessed January 2022).

